# The Medical Package Library

**Published:** 1915-04

**Authors:** F. E. Daniel


### EDITORIAL DEPARTMENT.

MRS. F. E. DANIEL, Publisher and Managing Editor.
DR. R. H. L. BIBB, Associate Editor.

CONTRIBUTING EDITORS:

Dr. H. Sheridan Baketel, New York, editor *Medical Times;* Dr. M. H. Boerner, Austin, Texas; Dr. G. Henri Bogart, Paris, Ill.; Dr. M. M. Carrick, Dallas, Texas; Dr. Edward H. Carey, Dean Medical Department, Baylor University, Dallas, Texas; Dr. W. L. Crosthwaite, Waco, Texas; Dr. T. D. Crothers, Hartford, Conn.; Dr. H. W. Cummings, Hearne, Texas; Dr. Oscar Dowling, New Orleans, President Louisiana State Board of Health; Havelock Ellis, M. D., London, Eng.; Dr. May Agnes Hopkins, Dallas, Texas; Dr. A. W. Fly, Galveston, Texas; Dr. G. B. Foscue, Waco, Texas; Dr. E. S. Goodhue, Holualoa, Kona, Hawaii; Dr. M. B. Grace, Seguin, Texas; Dr. Joe Gilbert, Austin, Texas; Dr. Charles H. Hughes, St. Louis, editor *Alienist and Neurologist;* Dr. James G. Kiernan, Chicago; Dr. George H. Lee, Galveston, Texas; Dr. John T. Moore, Houston, Texas; Dr. Frank Paschai, San Antonio, Texas; Dr. John Preston, Austin, Texas, Superintendent State Lunatic Asylum; Dr. G. Wilse Robinson, Kansas City, Mo.; Dr. Bacon Saunders, Fort Worth, Texas; Dr. Allen J. Smith, Philadelphia, Medical Department, University of Pennsylvania; Dr. Z. T. Scott, Austin, Texas; Dr. Ralph Steiner, Austin, Texas, President Texas State Board of Health; Dr. Walter S. Sutton, Kansas City, Mo.; Dr. A. E. Sweatland, Nacogdoches, Texas; Dr. John S. Turner, Dallas, Texas; Dr. Charles Venable, San Antonio, Texas.

---

### The Medical Package Library.

From time to time in recent issues, the TEXAS MEDICAL JOURNAL has announced its intention to arrange for the use of its subscribers a package library on medical subjects. This library, when completed, will consist of books, pamphlets, and articles bearing on practically every phase of medical diagnosis and treatment, grouped under proper headings and divisions. The purpose will be to assist busy practitioners in securing the best and latest available information on subjects in which they are interested, either in their practice or for the preparation of scientific medical papers.

It can readily be understood that a package library of the kind mentioned can never be complete, and that it will constantly be receiving additions. The TEXAS MEDICAL JOURNAL'S Package Library is yet largely in its formative stage, but the JOURNAL now stands ready to assist its subscribers by forwarding at an early date after the request, packages dealing with most of the subjects in which the physicians will be interested. These packages will be loaned without charge to subscribers desiring them, the borrowers merely paying express charges both ways and agreeing to return the packages within a reasonable time.

A number of physicians have written the editor of the JOURNAL that the system inaugurated by it will prove a great boon to busy physicians, and the sole purpose of the JOURNAL will be to render some valuable help to members of the medical profession. No profit whatever will be realized or expected from the undertaking beyond the appreciation and good will of those who avail themselves of it.

If you are especially interested in any medical or surgical problem and desire such assistance as the TEXAS MEDICAL JOURNAL can give through its Package Library, your requests will be given prompt attention.

MRS. F. E. DANIEL.

